# 50 States or 50 Countries: What Did We Miss and What Do We Do Now?

**DOI:** 10.1017/S1049023X20000746

**Published:** 2020-05-22

**Authors:** Frederick M. Burkle, Asha V. Devereaux

**Affiliations:** 1. Professor (Ret.), Senior Fellow & Scientist, Harvard Humanitarian Initiative, Harvard University & T.H. Chan School of Public Health, Cambridge MA Global Public Policy Scholar, Woodrow Wilson International Center for Scholars, Washington, DC USA Editor for Humanitarian Affairs, Prehospital & Disaster Medicine; 2. Pulmonary and Critical Care Medicine, Coronado, California USA

**Keywords:** Centers for Disease Control and Prevention, coronavirus, global public health, pandemics, population-based management, World Health Organization

## Abstract

There have been multiple inconsistencies in the manner the COVID-19 pandemic has been investigated and managed by countries. Population-based management (PBM) has been inconsistent, yet serves as a necessary first step in managing public health crises. Unfortunately, these have dominated the landscape within the United States and continue as of this writing. Political and economic influences have greatly influenced major public health management and control decisions. Responsibility for global public health crises and modeling for management are the responsibility of the World Health Organization (WHO) and the International Health Regulations Treaty (IHR). This review calls upon both to reassess their roles and responsibilities that must be markedly improved and better replicated world-wide in order to optimize the global public health protections and its PBM.

“*Ask a big enough question, and you need more than one discipline to answer it.*”

Liz Lerman, MacArthur “Genius” Fellow, Choreographer, Modern Dance legend, and 2011 Artist-in Residence, Harvard Music Department

## What Did We Miss?

There are marked differences in the case numbers and deaths between Washington State and New York State USA, both in numbers and management of COVID-19. But why?

Washington State from the outset supported public health “scientists” to make the population-based decisions to contain the spread, whereas New York State became mired in both political and economic decisions and pressures, admittedly as did other states, resulting in marked differences in disease transmission. Indeed, in many ways, the management of the pandemic differs markedly amongst the states, raising the question whether the United States is made up of 50 states or 50 countries from a public health perspective.^1^

These differences in management and outcomes are tied to early decisions required of all public health care systems leading the global effort to contain and manage any pandemic. The key to success is public health professionals who normally work behind the scenes in prevention, preparedness, vaccines, and control of infectious and environmental diseases. They often describe themselves as being the “invisible health profession,” yet as a profession, they are largely responsible for the majority of improvements in global life expectancy.^2^ Public health professionals, during the current pandemic, have silently assumed the leadership role in multiple countries and within the United States, as they do with all epidemics and pandemics. The functional and population-based approaches are complementary ways of understanding a complex problem, and both must be considered in preparedness and response to pandemics. The American Medical Association (AMA; Chicago, Illinois USA) defines population-based management (PBM) as an approach:

That allows one to assess the health status and health needs of a target population, implement and evaluate interventions that are designed to improve the health of that population, and efficiently and effectively provide care for members of that population in a way that is consistent with the community’s cultural, policy, and health resource values. [It] does not detract from individuality, but rather adds another dimension, as individuals benefit from the guidelines developed for the populations to which they belong.^3^

The PBM decisions during public health emergencies (PHEs), such as SARS Co-V2, are a core function of public health law. These decisions shift emphasis from individual care to population-based decisions, while recognizing limited or absent resources to ensure the best possible outcome for society as a whole. Public health leadership is judged by their capacity and capability to understand the consequences of such population-based decisions, and their capacity to operationalize coordinated investigations and analyses, resulting in eventual control of the spread of the disease. In implementing PBM, public health professionals may find themselves in a situation in which the demand for resources exceeds supply, requiring coordinated responses across many disciplines, the capacity to harness proper national and global public health expertise, infrastructure, epidemiological and scientific analysis, and develop preventive measures that are compatible with population density, adequate public health protections, both the appropriate and inappropriate use of medications and procedures, and unexpected challenges provoked by the virus itself. When deployed appropriately, PBM can help unify instead of divide a country.

Without PBM and PHE leadership in management decisions, maximizing survival opportunities during the COVID-19 pandemic often failed. The PBMs are formed as interdisciplinary and multidisciplinary integrative expertise (anthropologists, sociologists, epidemiologists, attorneys, clinical medicine and nursing, pharmacy, industry, technology, etc.) who reflect the reality of current field demands and health crisis management requirements that define the working relationships and uncomfortable but real decision making. Health Crisis Managers, within the PBM framework, emphasize “careful planning, protocol and procedure writing, team member selection, training, and practice among the multidisciplinary team.”^4^ A nimble team that is capable of adapting to the crisis situation is the key to success. In a novel pandemic, plans and protocols will require continued modification based upon evolving science and data, as well as rapid communication of decisions made as well as lessons-learned. Accountability and transparency are vital components. The compatibility of these concepts depends on what one is used in administrating triage decisions, which weigh the increase in the likelihood of medical success, epidemic control, and conservation of scarce resources.^5,6^ This is what public health managers see and act upon in the daily epidemiological data. The PBM structures aim to improve the health of an entire population and include health outcome data of individuals, including the distribution of resources within the population. Strategies on how to deliver medical care to all populations during pandemics are planned events decided within the organized and multidisciplinary PBM structure. The PBM also initiates triage categories, a complex topic, with which most clinicians have limited experience, that often causes difficulty when one makes the shift from individual patient triage to population-based triage. In Italy, a pandemic triage protocol previously debated and decided upon, which included a non-survivable category, came to fruition when ventilators were no longer available.^5^ Proper anticipated PBM decisions mitigate the gravity of such decisions away from the individual health care provider who may not be aware of the larger system-wide limitation of resources.

Global leaders in the PBM process include the World Health Organization (WHO; Geneva, Switzerland) and the US Centers for Disease Control and Prevention (CDC; Atlanta, Georgia USA). The WHO is a United Nations agency that answers to an annual assembly of the world’s health ministers. The WHO convenes panels of independent global experts. It has been criticized for being too rigidly bureaucratic, authoritarian, inflexible, and plagued by limited funding.^7,8^ The CDC is a branch of the United States government and answers to the President, Congress, and the courts. The CDC makes recommendations based on advice from internal experts.^9^ It too has suffered with senior scientists lodging ethics complaints alleging the federal agency is being influenced by outside corporate and political interests,^10^ severe budget cuts, and that the science-based approach is being ignored and silenced because of increasing politicization of the CDC’s work. The side-lining of the CDC has left the impression that they have “almost disappeared from the national conversation.”^11^ A shining star in the CDC history is the Epidemic Intelligence Service (EIS), founded in 1951, producing “disease detectives” whose alumni have gone on to take key public health roles world-wide.

Despite the existence of studied guidelines from prior pandemics, the COVID-19 tragedy response has proven fragmented, highly political, and lacks the cross-disciplinary understanding of what and how public health crises translate into operational cooperation before, during, and after a pandemic. There is no question that the CDC did lose precious time and trust of the US population by their delay in testing and weakened personal protective equipment (PPE) recommendations, which resulted in political intervention in the US. This has stripped the CDC of its once robust preparedness and response capability and capacity.^12^ Unfortunately, similar interferences have plagued the WHO and its International Health Regulations Treaty (IHR),^13,14^ once designed to “prevent, protect against, control, and provide a public health response to the international spread of disease in ways that are commensurate with and restricted to public health risks, and which avoid unnecessary interference with international traffic and trade.”^15^

A constant impediment has been the lack of what defines a PHE, which is defined as much by its public health consequences as by its causes and precipitating events, all of which have the potential to overwhelm routine community capabilities to manage them.^16^ Within the WHO and by public health professionals, they are defined as:

An occurrence or imminent threat of an illness or health condition, caused by bioterrorism, epidemic or pandemic disease, or (a) novel and highly fatal infectious agent or biological toxin, that poses a substantial risk of a significant number of human facilities or incidents or permanent or long-term disability.^17^

The declaration of a state of PHE permits the governor to suspend state regulations and change the functions of state agencies. In these definitions, PHEs are defined through public health imperatives. Political and economic leaders, on the other hand, frequently define a PHE through both political and economic imperatives, leaving public health and governmental decision makers often talking past each other, causing considerable confusion and inappropriate decision making. This has become incrementally worse, especially in more autocratic and authoritarian regimes.^14^ The purposeful lack of, or interference from, political leadership decisions have resulted in increased transmission of disease and undermine necessary population-based decisions that otherwise would lead to control of the pandemic.^18^

Additionally, traditional health care systems care for patients individually, while public health is about caring for an entire population. The bevy of health care professionals responding to a pandemic come from academic training institutions that emphasize one-on-one patient skills to diagnose, treat, and prevent injuries and illnesses in individual patients. Even in the increased provisions for team-based health care, all work collaboratively with an individual patient to ensure that the physician alone will make “sound, individual, clinical decisions.”^19^ The nuances of PBM care is rarely included in their training.

The incidence of zoonotic diseases that spread from a reservoir animal to a human-animal such as H1N1, MERS, SARS, Ebola, and COVID-19 have increased globally from 20% decades ago to more than 71% of new diseases discovered today. Their impact has been exacerbated or accelerated by climate change extremes, biodiversity losses, rapid unsustainable urbanization, and increasing scarcity of food, water, and energy for many populations.

The communicable disease will classically be silent, odorless, invisible, and immediately undetectable. Severity is gauged by the ability of the disease to infect and transmit itself in a susceptible population, is primarily managed by outbreak investigation and control involving a collection of interventional tasks designed to identify and terminate human-to-human transmission, control the pandemic, and ultimately save the maximum number of lives. Infectious disease outbreaks have the uncanny capacity to question the status quo, catalyze smoldering unrest, and most importantly, reveal population-based public health imperfections.^20,21^ Yet, the most challenging task for responders is often the demands made in moving from individual to population-based care.^5,22^,

Population-based management must focus on the system established to improve the health outcomes of a population, including the distribution of such outcomes within the population. This involves:^23^

1. Key Elements of Preparedness:
  - Health risk assessment;
  - Legal climate (primarily legal authority and liability);
  - Roles and responsibilities;
  - Incident Command System;
  - Public engagement;
  - Epidemiology functions;
  - Laboratory functions;
  - Countermeasures and mitigation strategies;
  - Mass health care;
  - Public information and communication; and
  - Robust supply chain. As well as,
2. Expert and Fully Staffed Workforce:
  - Operations-ready workers and volunteers; and
  - Leadership (train and develop public health leaders). Along with,
3. Accountability and Quality Improvement:
  - Testing operational capabilities;
  - Performance management; and
  - Financial tracking.

Whereas the PBM approach requires a departure from the individual care role of clinicians with patients, this does not minimize the importance of clinical responsibilities, but rather adds the dimension of new public health and surge-capacity interventions that improve access and availability of limited health resources for the entire population.^24^ All individuals share the following:^5^

- All either have the same condition or are susceptible to it;
- All have shared health care needs;
- All require some intervention;
- All fall into one of the triage management categories; and
- Pandemics may require a sustained operational response lasting 12-24 months or longer.

No one is excluded. However, the devil is in the details, especially when these mandates are dismissed or ignored by politicians and economic leaders. This is exemplified by numerous instances of private health care institutions dismissing health care providers for revealing serious lapses in PPE to superiors and/or the local media. Many essential critical workers have been disproportionately affected by COVID-19 either due to weakened PPE, or early inadequate testing of patients, or both. Both population-based medicine and patient-centered medicine “should be perfectly aligned. And they almost are.”^3^

The only proven treatment options to stem the spread of most viral diseases are social distancing strategies (SDS), some anti-viral medications, and vaccines. Unfortunately, SDS which involves community quarantines, isolation, or lockdowns are too often started late, prematurely ceased, or include many exceptions or conditions risking maximization of their efficiency or any valid measurement of success.^25^ Similarly, contact tracing, screening, and extensive testing as key strategies for preventing further spread have also proven to be inadequate and inconsistent in the US states.^25^ This has resulted in a disparate response in each US state driven by differing political, economic, scientific, or social factors and causing us to feel as if we may have 50 different countries.

## What Do We Do Now to Save Global Public Health: and Thereby Save Ourselves?

Our biggest fear is that recovery from COVID-19 will foster no major change in how the world prepares for and responds to epidemics and pandemics. That is quite possible as denial is the strongest of human defenses. We must, as health care professionals, strongly support the recognition that global public health crises will continue unless there is full acceptance of the multiple global insults that help catalyze these infectious disease events, including climate change, biodiversity loss, deforestation, and rapid unsustainable urbanization, as conditions that support survival and expansion of reservoir animals.

Science is being ignored, especially by authoritarian and autocratic regimes, because they cannot challenge science and will forcefully ignore it or try to alter its message. The individual State Departments of Health are run by political appointees and differ greatly in capability, capacity, and containment decisions; whereas some Departments are excellent models of efficiency, others focus primarily on chronic diseases demonstrating limited capacity in infectious disease epidemiology, PBM, or working in a coordinating manner with CDC during previous epidemics.

The WHO and IHR, organized to manage population-based diseases, have failed to meet population-based expectations, in part due to influence from powerful political donors that the WHO has become dependent on for its financial existence. The “Collective Independent WHO,” a grassroots movement was first to demand WHO’s independence to fulfill its duties to protect populations affected by radioactive contamination after Chernobyl.^26^ It is equally relevant to place pressure on WHO to fulfill its duty to achieve “the attainment by all peoples of the highest level of health” in all matters. This was denied to Taiwan when the People’s Republic of China, with its “great power status and permanent seat on the UN Security Council,” prevented WHO from replying to Taiwan’s early call for assistance for concerns over COVID-19 human to human transmission, and was accused of “putting politics first.”^27^ The WHO’s “exclusion of Taiwan from the global fight against the pandemic is a reckless dereliction of duty.”^28^ To be effective globally, WHO must be totally independent as a treaty-based organization, sanctioned and fully funded by the UN and all its members, without restrictive conditions or favoritism. It cannot be dependent on outside financial assistance to do its work. The WHO must be the sole public health authority unencumbered by all political or economic attempts to do otherwise.^14^ As such, besides expanding WHO assets within Geneva Headquarters, substantial replication of WHO capabilities must occur at all six WHO Regional Organizations.

Currently, WHO exists separately from the US-dominated CDC, which conducts public-health-oriented research, produces and supplies diagnostic reagents, and assists with outbreak investigations, technical assistance, and capacity building, including laboratory and epidemiological training to strengthen the WHO outbreak control. Together with CDC, WHO Collaborating Centers for surveillance, epidemiology, and control of infectious diseases work together on a variety of activities. Through a new global public health model, global surveillance and response systems would be expanded regionally and nationally.^29,30^ Every country and/or region must have a CDC to supplement the US regional CDCs, those in China, the European CDC in Stockholm, and the new African CDC which serves as a specialized technical institution of the African Union member States. All of these assets would be reorganized and financially supported as a wheel and spoke model designed to work in smaller teams in support of PBM, which can scale upwards and build individual country capacity and value more rapidly. Collaboration among setting competency-based training and people-power initiatives, especially with closed off and insulated authoritarian countries, would be among some of the most challenging demands that would redefine global public health, and expanding operational PBM skill-sets from the smallest of the countries to the global population.

Furthermore, the global public health community cannot tolerate the increasing political interference that authoritarian and autocratic regimes have exercised over WHO. In a highly integrated globalized world, both the WHO with its Treaty have the potential to become one of the most effective mechanisms for crisis response and risk reduction world-wide.^14^ The world’s health practitioners, especially public health decision makers, must break their silence and collectively advocate for a stronger IHR Treaty, and a return of the WHO’s singular global authority that supports a highly coordinated, multidisciplinary, and science-based PBM.

Most importantly, public health across the entire global population can no longer be recognized as the “invisible profession” as it assumes ownership as the sole public health authority. This applies to all populations as well as being supported by the health societies and the governments they live in.

The global medical and public health curriculum must include zoonotic diseases as a priority in education and health care delivery. Medical and public health efforts must not be directed at the response phase alone in the disaster cycle, but must capture operational and research responsibility across the entire disaster cycle: prevention, preparedness, response, recovery, and rehabilitation, and support new academic programs that train scientists and health crisis managers.^31^ Academic initiatives currently arising out of Europe will offer credentialed Master’s and PhD-level programs to meet expected leadership roles in support of WHO’s and IHR’s expanding global initiatives.

Public health and public health infrastructure and systems in developing countries must be viewed as strategic and security issues that deserve international public health resource infrastructure and monitoring, again covering the entire disaster cycle. All six WHO Regional Offices must have similar multidisciplinary professional assets in support of zoonotic sciences, which would be further resourced from WHO global assets during any epidemic or pandemic. The goal is to identify and begin the containment process as rapidly as possible!

In 2016, WHO completed the Joint External Evaluation (JEE) of IHR core capacities of the United States. This Mission Report was an evaluation of the US’s capacity to prevent, detect, and rapidly respond to public health threats.^32,33^ The assessment used the WHO-IHR JEE tool and reported that whereas a “One Health” approach is utilized in the evaluation and good collaboration was noted between and within the states, the government and other stakeholders favor health strategies that have a broader ownership at the federal, state, and local levels.^32^ It identified challenges that might arise and encouraged the country to continue to reinforce and develop collaboration across all levels of the public health system to strengthen IHR (2005) core capacities. The WHO cautioned that the apparent attrition in senior-level expertise across a range of core capacities was evident and encouraged recruitment and retention of personnel. They further recommended that health workforce models are needed that are adaptable to local circumstances, local risk assessments, take into account the ability of some personnel to have multiple qualifications, and that multidisciplinary experts were needed from relevant health sectors and specialists in different emergency situations.^34^

Unfortunately, the 2020 coronavirus response revealed little correlation to the JEE recommendations. The JEE report failed to deal with the impact of federal systems in large countries like the US, China, and Russia, where evaluations must be done differently than the “One Health” approach, where travel restrictions were more easily curtailed in smaller countries than in larger countries where travel and daily business transactions are the life-blood of their international political and economic power.^34^ This led to a lack of immediate leadership from state-level public health experts who have had to yield to early political and economic control over basic public health decisions. Too often this has resulted in fragmented decisions and mistakes across all states, their artificial borders, and are described to be “out of control of WHO or even national health authorities” to correct.^35^ As such, the US states too often performed as though they were 50 different countries.

Once thought impossible, post-WWII survivors cooperatively accomplished the daunting task of rebuilding a better world. Today, with over four million COVID-19 cases and over 281,000 deaths, the world community is facing similar challenges, doubts, and fears. This is not the first attempt to advocate for WHO to “complete and restore the original mandates as a collaborative and coordinated global network responsibility, not one left to the actions of individual countries.”^13^ Each and every global and national organizations will face challenges to reform their global public health roles and responsibilities so there is consistency in public health protections across all borders separating individual states within the US or separate countries. Any global public health collective must begin now to think and plan for a new generation of public health care managers and scientists who are trained across the entire disaster cycle to anticipate and manage population-based crises resulting in epidemics and pandemics as well as nuclear war, climate change, and biodiversity loss, which alone that can lead to global devastation.

A former CDC-EIS officer, “dismayed with seeing the communication principles that the CDC had honed over the years being disregarded,” is credited with being instrumental in convincing Washington State to “scrupulously follow proven EIS protocols,” saving the state from the rapidly rising case and death rates seen in New York and other states.^36^ Connecticut US Senator Chris Murphy underscored the lack of consistency and quality with CDC guidance, calling that provided on reopening of states as “criminally vague.”^37^ The EIS model needs to be strengthened and further replicated world-wide to fuel the expertise and inventiveness of a future WHO/IHR and CDC partnership that serves global public health and PBM, both globally and in its replication in individual countries. There is no excuse!
